# Zoonosis: a comprehensive database of zoonotic pathogens

**DOI:** 10.1093/database/baaf062

**Published:** 2025-10-15

**Authors:** Boyuan Zhang, Dan Liu, Weiwen Wang, Xiaoqiang Li, Yifei Xie, Huitong Li, Hongmei Zhou, Jianshuai Gao, Hui Jiang, Xuezheng Fan, Jiabo Ding, Qingchun Shen, Xizheng Ma

**Affiliations:** Key Laboratory of Animal Biosafety Risk Prevention and Control (North), Ministry of Agriculture and Rural Affairs, Institute of Animal Science, Chinese Academy of Agricultural Sciences, No. 2 Yuanmingyuan West Road, Beijing 100193, China; Key Laboratory of Animal Biosafety Risk Prevention and Control (North), Ministry of Agriculture and Rural Affairs, Institute of Animal Science, Chinese Academy of Agricultural Sciences, No. 2 Yuanmingyuan West Road, Beijing 100193, China; BGI Research, Beishan Road, Yantian District, Shenzhen 518083, China; Guangdong Provincial Genomics Data Center, BGl Research, Beishan Road, Yantian District, Shenzhen 518083, China; BGl-Wuhan Research, Kejisan Road, Donghu District, Wuhan 430074, China; Key Laboratory of Animal Biosafety Risk Prevention and Control (North), Ministry of Agriculture and Rural Affairs, Institute of Animal Science, Chinese Academy of Agricultural Sciences, No. 2 Yuanmingyuan West Road, Beijing 100193, China; Key Laboratory of Animal Biosafety Risk Prevention and Control (North), Ministry of Agriculture and Rural Affairs, Institute of Animal Science, Chinese Academy of Agricultural Sciences, No. 2 Yuanmingyuan West Road, Beijing 100193, China; MGI Tech Co., Ltd (BGI Manufacturing), Beishan Road, Yantian District, Shenzhen 518083, China; Key Laboratory of Animal Biosafety Risk Prevention and Control (North), Ministry of Agriculture and Rural Affairs, Institute of Animal Science, Chinese Academy of Agricultural Sciences, No. 2 Yuanmingyuan West Road, Beijing 100193, China; Key Laboratory of Animal Biosafety Risk Prevention and Control (North), Ministry of Agriculture and Rural Affairs, Institute of Animal Science, Chinese Academy of Agricultural Sciences, No. 2 Yuanmingyuan West Road, Beijing 100193, China; Key Laboratory of Animal Biosafety Risk Prevention and Control (North), Ministry of Agriculture and Rural Affairs, Institute of Animal Science, Chinese Academy of Agricultural Sciences, No. 2 Yuanmingyuan West Road, Beijing 100193, China; Key Laboratory of Animal Biosafety Risk Prevention and Control (North), Ministry of Agriculture and Rural Affairs, Institute of Animal Science, Chinese Academy of Agricultural Sciences, No. 2 Yuanmingyuan West Road, Beijing 100193, China; Key Laboratory of Animal Biosafety Risk Prevention and Control (North), Ministry of Agriculture and Rural Affairs, Institute of Animal Science, Chinese Academy of Agricultural Sciences, No. 2 Yuanmingyuan West Road, Beijing 100193, China; BGI Research, Beishan Road, Yantian District, Shenzhen 518083, China; Guangdong Provincial Genomics Data Center, BGl Research, Beishan Road, Yantian District, Shenzhen 518083, China

## Abstract

Emerging infectious diseases pose a significant threat to global public health and economic security, with zoonoses accounting for a substantial proportion. Livestock such as cattle and sheep are critical reservoirs for zoonotic pathogens and play a key role in transmitting these pathogens to humans and other animals, including dogs and wildlife, due to their close interaction with diverse populations. In this study, we introduce Zoonosis (http://zoonosis.cn/zoonosis/), a comprehensive database that integrates data on pathogens, including *Brucella, Mycobacterium tuberculosis*, and *Bacillus anthracis*. Currently, Zoonosis integrates over 4500 samples from more than 60 countries, with a total data volume of 1.8TB, providing a global perspective on zoonotic disease distribution. Equipped with user-friendly visualization and analysis tools, Zoonosis enables rapid biological data interpretation, aiding disease diagnosis and prevention. This resource supports virology, zoology, and epidemiology experts in monitoring cross-species transmissions and mitigating future zoonotic outbreaks.

**Database URL**: http://zoonosis.cn/zoonosis/

## Introduction

In recent years, the increasing frequency and expanding impact of zoonotic diseases have been driven by global ecological changes, shifts in human lifestyles, and enhanced population mobility [1, 2]. Over 200 known zoonoses—including influenza, tuberculosis, anthrax, and brucellosis—pose significant threats to human health and socioeconomic stability [3–5]. Notably, 60% of emerging infectious disease outbreaks in the past seven decades originated from animal-derived pathogens [6–8]. Concurrently, intensified global travel, rapid urbanization, and climate change are dynamically reshaping epidemic patterns, underscoring the critical need for interdisciplinary research into zoonotic transmission mechanisms, prevention strategies, and global health security [9–11].

Livestock, particularly cattle and sheep, serve as major reservoirs for zoonotic pathogens due to their close interaction with humans [12, 13]. Species such as cattle, sheep, horses, and dogs can harbour and transmit pathogens responsible for tuberculosis, brucellosis, anthrax, and rabies, posing substantial public health risks. Brucellosis demonstrates elevated prevalence rates in Africa and Asia, while persisting as a veterinary and public health challenge even in developed European countries [14, 15]. These realities highlight the urgency of establishing a comprehensive global zoonotic disease repository to identify novel pathogens, detect interspecies spillover risks, and map pathogen diversity, geographic distribution, and host/vector specificity. These efforts are critical for risk assessment and the development of targeted prevention strategies against future epidemics [9].

Here, we introduce a novel zoonotic disease database, designated as Zoonosis (http://zoonosis.cn/zoonosis/). This platform consolidates comprehensive data on pathogens such as *Brucella, Mycobacterium tuberculosis*, and *Bacillus anthracis*, establishing a robust online resource for zoonotic disease research. Additionally, we have developed user-friendly online visualization tools to facilitate diverse comparative analyses. These tools enable scientists to intuitively explore and compare pathogen genomes, rapidly identify pathogen species, and support pathogen diagnosis and epidemiological investigations. Collectively, this initiative advances multidisciplinary research on zoonotic pathogens, enhancing capabilities for outbreak prevention and control. It is very meaningful to establish this Zoonosis platform, since *Brucella, M. tuberculosis*, and *B. anthracis* are the most important bacterial zoonoses.

## Materials and methods

### Data curation

Zoonosis compiles information on *Brucella, M. tuberculosis*, and *B. anthracis*. The database includes pathogens associated with four mammalian hosts—sheep, cattle, dogs, and pigs—and encompasses over 1.8 TB of data.

To retrieve the sequences and assembly data of *Brucella, M. tuberculosis*, and *B. anthracis*, we conducted an exhaustive search in the Sequence Read Archive [16] and Assembly modules of the National Center for Biotechnology Information (NCBI) database (https://www.ncbi.nlm.nih.gov/), using the keywords ‘*Brucella*’, ‘*M. tuberculosis*’, and ‘*B. anthracis*’. To ensure data integrity, detailed metadata were recorded during data collection, including pathogen name, host vector, sample location, specific latitude and longitude, collection date, isolation source, and other relevant information. The collected data were organized into projects, with metadata displayed in the project and sample modules based on the CNGB Sequence Archive (CNSA) platform (https://db.cngb.org/cnsa/) provided by our partner, Shenzhen National Gene Bank. CNSA (China National GeneBank Sequence Archive) is a biological data archiving and management platform provided by the Shenzhen-based China National GeneBank.

We conducted a meticulous review of these records, correcting inconsistencies and incomplete content based on relevant literature. Data with insufficient volume or significant sequence length discrepancies were excluded.

### Database construction

The backend of Zoonosis comprises the database management system and high-performance storage solutions, utilizing network-attached storage (NAS) and distributed storage systems such as Lustre to support data submission, archiving, and quality control for data analysis. NAS is a system that facilitates high-performance data storage and access through network connectivity. The frontend of Zoonosis is developed using the Django framework (https://docs.djangoproject.com/; v4.2.10), which includes various built-in web development tools and follows the model-view-controller architecture pattern, providing a user-friendly interface. Additionally, the independent Basic Local Alignment Search Tool (BLAST) [17] (https://ncbiinsights.ncbi.nlm.nih.gov/2021/07/09/blast-2-12-0/; v2.12.0), Jbrowse [18] (https://jbrowse.org/jb2/; v1.16.4), and EBISA programs are integrated into the website, enabling users to analyse pathogen gene and protein information within Zoonosis. BLAST is a sequence alignment tool for bioinformatics that rapidly identifies sequences with high similarity to query sequences within large-scale databases. Finally, we integrated a strain phylogenetic analysis pipeline, allowing users to leverage Zoonosis datasets in conjunction with the GTDB-Tk software (https://ecogenomics.github.io/GTDBTk/index.html; v2.4.0) and ITOL platform (https://itol.embl.de/; v7) for comprehensive evolutionary investigations. Comprehensive security measures are implemented across all layers, including firewalls, Secure Shell (SSH) key-based authentication, regular system updates, application-level protections (such as CSRF, SQL injection, and XSS prevention), secure coding practices, regular data backups, disaster recovery planning, and strict access control with audit logging. This architecture ensures efficient, secure, and scalable management of zoonotic pathogen data and related analysis workflows.

## Results

### Overview of zoonosis

Zoonosis is publicly accessible through a highly intuitive and responsive web interface (http://zoonosis.cn/zoonosis/). Zoonosis also provides basic tools to browse the data of any single pathogen sample. The database classifies the data by pathogen and species to form thematic projects. Within each thematic project, the CNSA-based project module can display project information, sample information, etc., and relevant data can be searched using keywords or query sequences. In order to provide the scientific community with an overall picture of the spatial and temporal diversity and dynamics of zoonotic pathogens and vector transmission, the spatial distribution of pathogens has been added. In addition, we also hope to embed basic analytical tools for zoonotic disease research in Zoonosis.

The Zoonosis provides basic search services. In the Participation module, data can be searched by experimental data number, species classification, platform, and sample name. Detailed information on pathogen data can be obtained by combining with the CNSA project display page. At the same time, the spatial distribution of pathogens reflects the overall spatial and temporal diversity and dynamics of zoonotic pathogens and vector transmission. In the research module, users can choose to view the data set of pathogens in different species. In addition, in the tool module of the Zoonosis, the BLAST online tool provides comparison data selection of pathogen genomes, coding sequences (CDS), protein sequences, etc., and combines query sequences to quickly perform data analysis. In addition, in the JBrowse tool, genome data of different pathogens can be selected to facilitate the display of gene data.

So far, the Zoonosis has collected more than 4500 samples, with a data size of 1.8TB, including more than 10 assemblies of three pathogens. Among them, more than four mammalian hosts are involved, and the sample sources are more than 60 countries (Table 1). At the same time, detailed information is provided for these data.

**Table 1.** Statistics of the related information of pathogens collected in the Zoonosis (as of September 2024).

Germs/diseases	Experiment count	Data size	Assembly quantity	Host species	Number of countries sampled
*Brucella*	2515	741.54GB	5	60	67
*Mycobacterium tuberculosis*	21	5.57GB	1	1	2
*Bacillus anthracis*	2113	1112.63GB	1	16	60

### Data analysis tools of zoonosis

We provide three basic tools for zoonotic bacteria research, including BLAST (for gene sequence alignment and similarity search), JBrowse (for genome browsing and visualization), and EBISA (for rapid sequence identification of unknown strains).

JBrowse genome browser allows for intuitive exploration of genomes and their associated large-scale datasets. The tool can provide the genome of pathogenic bacteria and start JBrowse through this module to visualize the selected genome. At the same time, in the JBrowse browsing interface, the genome can be viewed through function keys such as sliding and zooming. Zoonosis is embedded with visualization tools for displaying and exploring genomes and other biological data. In bioinformatics, BLAST (Basic Local Alignment Search Tool) is a program used to perform alignments of primary biological sequence data. Users can submit amino acid sequences of proteins or nucleotide sequences of DNA and align them against the genomes or CDS sequences of pathogenic bacteria. We can gain an in-depth understanding of the biological characteristics of the target sequence and conduct subsequent experimental verification and research work based on this. EBISA is a tool for rapid sequence identification of unknown strains. After downloading and installing, users can start the program and select the uploaded sequence for analysis. It can determine whether they belong to 18 bacterial strains, including *B. anthracis, Yersinia pestis, B. abortus, B. suis, B. melitensis, B. ovis, B. canis, Clostridium tetani, Bordetella pertussis, M. leprae, Chlamydia trachomatis, Neisseria meningitidis, M. ulcerans, Listeria monocytogenes, Mycoplasma pneumoniae, Vibrio vulnificus, Legionella pneumophila*, and *Streptococcus suis*. These tools empower scientists to intuitively explore and compare pathogen genomes, rapidly identify pathogen species, and facilitate pathogen diagnosis and epidemiological research, thereby advancing the study of zoonotic pathogens in a comprehensive manner.

### Modules of zoonosis

Zoonosis integrates three core modules—literature, patents, and products—to meet diverse user needs. The literature module compiles academic journals, research reports, conference papers, and other resources related to zoonotic diseases, covering various aspects such as pathogen research, diagnostic technologies, vaccine development, epidemiological investigations, and prevention strategies. It supports full-text downloads to facilitate rapid access to academic resources. These research outcomes provide scientific foundations and technical support for animal disease control, pathogen biological characterization studies, vaccine development, public health security, and molecular biology research. The patent module aggregates patent information across multiple domains, including pathogen detection, vaccine development, diagnostic marker discovery, microbial culture identification, and genetic engineering. It offers patent previews and downloads to assist enterprises and individuals in tracking technological innovation trends and mitigating infringement risks. The product module delivers market data on zoonotic disease-related products, aiding users in market analysis, and product planning. The membership module highlights principal contributors of patents, publications, and key members of research teams, providing their contact information to foster collaboration and communication.

By integrating these three functional modules, Zoonosis not only offers researchers efficient access to academic resources but also delivers decision-making support for enterprises and government agencies. Additionally, it serves as a public knowledge platform for understanding and preventing zoonotic diseases.

### Comprehensive comparison and analysis

The Zoonosis provides a detailed overview of zoonotic and vector-borne pathogens across four major mammalian hosts (sheep, cattle, dogs, and pigs). It features a zoomable global map with gradient colour scales to visualize pathogen data distribution by country/region, enabling intuitive global comparisons. Studies reveal connections between pathogens and hosts, with host/vector diversity reflecting pathogen adaptability. Spatial and temporal correlations between zoonoses, host diversity, and environmental changes allow for comparative analysis of zoonotic and vector-borne pathogens across different regions and timeframes. The data on the species and host distributions of *Brucella, M. tuberculosis*, and *B. anthracis* bacteria reveal several notable findings, each with its own research significance.

**Figure 1.**
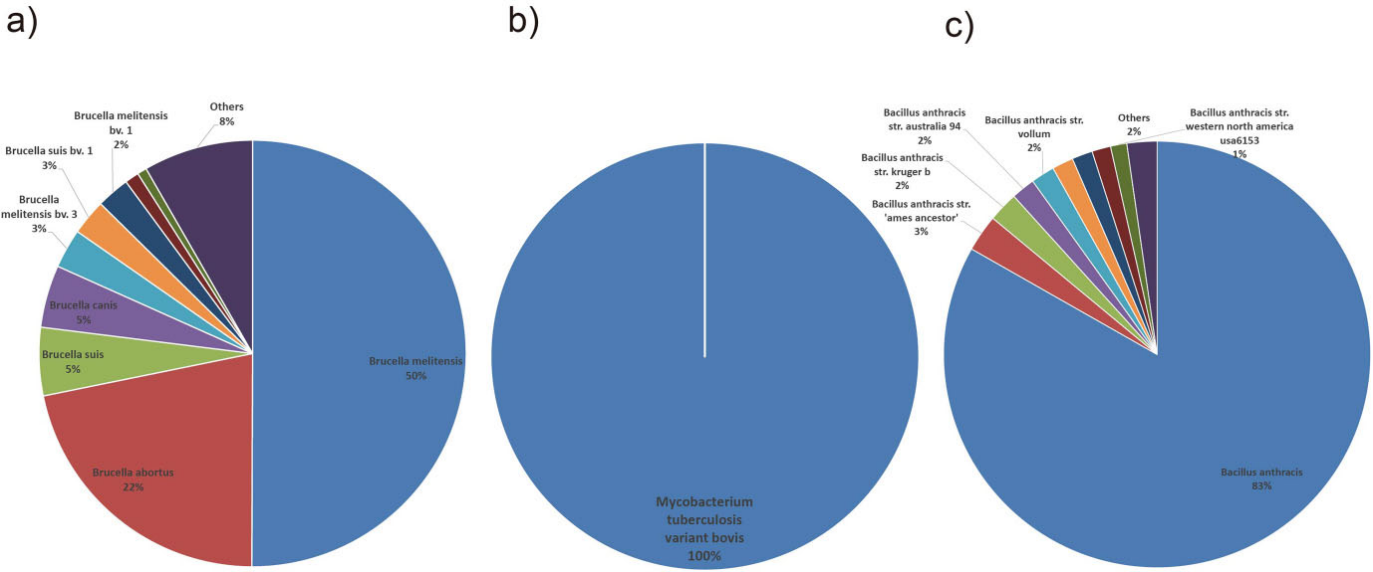
Species distribution of bacterial pathogens. (a) *Brucella* species distribution: *B. melitensis* (58%) and *B. abortus* (22%) dominate. (b) *Mycobacterium tuberculosis* species distribution: *M. tuberculosis variant bovis* accounts for 100% of detected species. (c) *Bacillus anthracis* species distribution: *B. anthracis* and other species account for 85%, indicating strain diversity.

**Figure 2.**
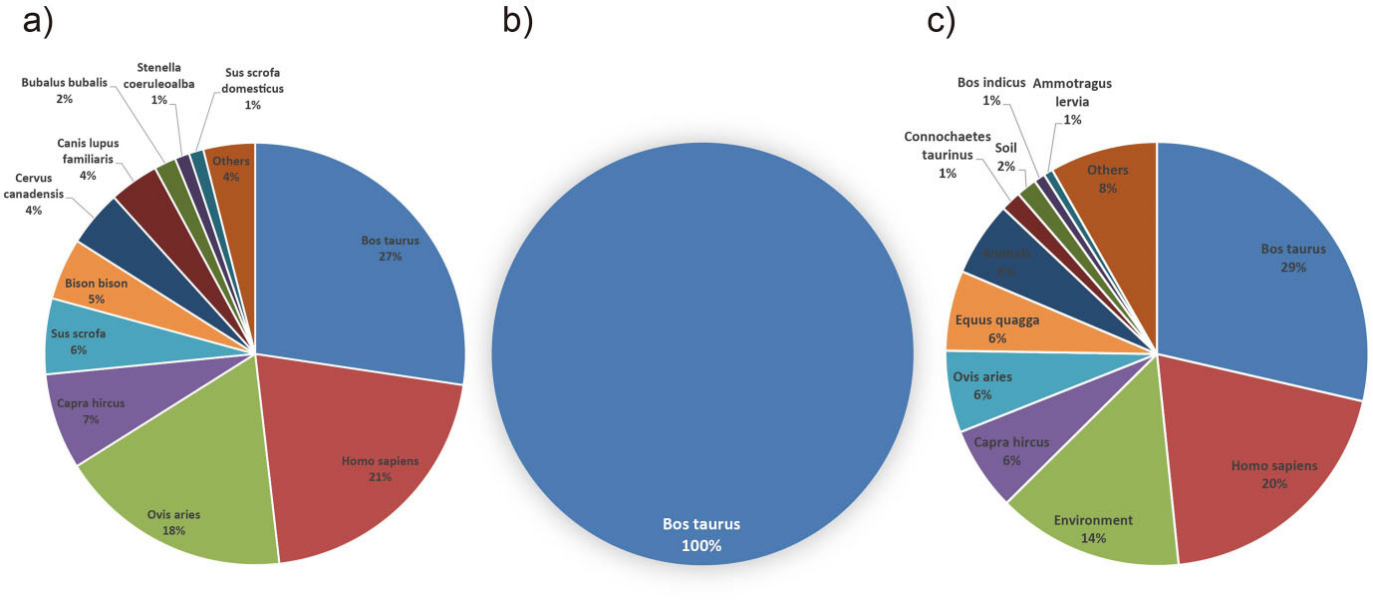
Host distribution of bacterial pathogens. (a) *Brucella* hosts: *Bos taurus, H. sapiens*, and *O. aries* are the primary hosts, reflecting zoonotic transmission routes. (b) *Mycobacterium tuberculosis* hosts: *Bos taurus* dominate, indicating limited host range. (c) *Bacillus anthracis* hosts: *H. sapiens, Bos taurus*, and environmental sources are key, showing broad host adaptability.

For *Brucella* bacteria, the species distribution shows a high diversity, with *B. abortus* being the most prevalent, accounting for 58% of the total, followed by *Brucella melitensis* at 22%. The presence of multiple subspecies and biovars, as well as rare or environment-specific species, highlights the adaptability and widespread nature of *Brucella* in various hosts and environments. The host distribution further supports this, with *Homo sapiens* (humans), *Ovis aries* (sheep), and *Bos taurus* (cattle) being the top hosts. This information is crucial for understanding the epidemiology of *Brucella* infections, as it helps identify the primary hosts and potential transmission routes. Research into the species and host distributions of *Brucella* can aid in the development of targeted prevention and control strategies, as well as the design of effective vaccines and therapeutic agents (Fig. 1a, Fig. 2a) [19].

In contrast, the species distribution of *M. tuberculosis* is relatively homogeneous, dominated by *M. tuberculosis variant bovis*, which accounts for 100% of the species detected in this category. The host distribution confirms this, with *Bos taurus* (cattle) being the primary host. Studying the species and host distributions of *M. tuberculosis* is essential for understanding the transmission dynamics between animals and humans, and for developing effective control measures to prevent the spread of *M. tuberculosis* to humans and other animals (Fig. 1b, Fig. 2b).

For *B. anthracis, B. anthracis* and a diverse range of strains categorized as ‘Others’ together account for 85% of the species distribution. The host distribution shows that *H. sapiens* and *Bos taurus* (cattle) are the top hosts, followed by the environment, indicating that *B. anthracis* can affect both humans and animals, as well as persist in the environment. Research into the species and host distributions of *B. anthracis* is critical for biosafety and biodefense, as *B. anthracis* is a highly lethal and stable pathogen (Fig. 1c, Fig. 2c).

**Figure 3.**
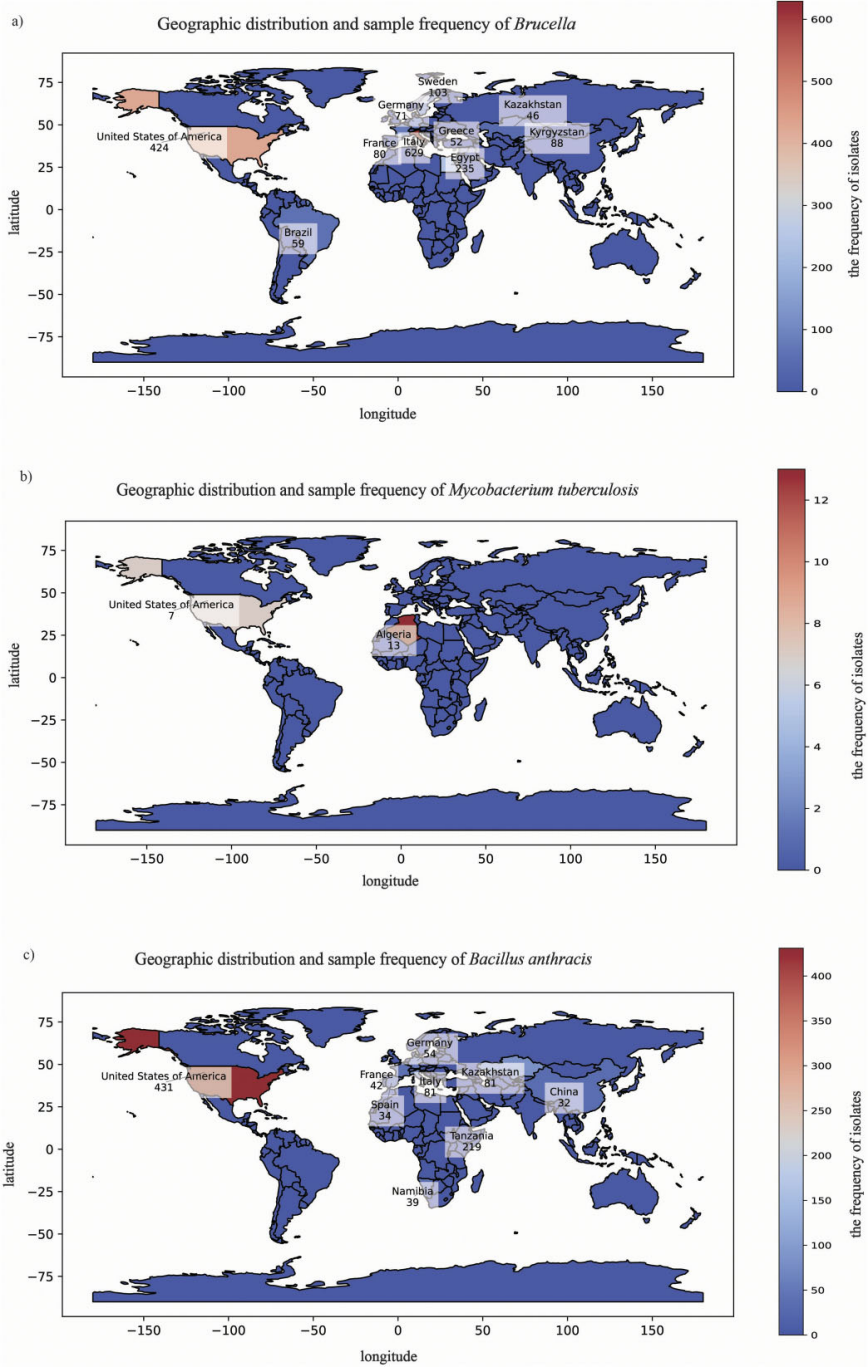
Global distribution of bacterial pathogens. For each panel, the world map displays the number of isolates collected from each country. The fill of each country represents isolate frequency on a continuous scale. The colour bar provides a quantitative reference for frequency values, with the maximum value corresponding to the country with the highest number of isolates in each panel. Panel (a) shows *Brucella* isolates, which are widespread across multiple regions, with higher frequencies in Italy (629), the USA (424), and Egypt (235). Panel (b) shows *M. tuberculosis* isolates, which have a limited distribution, primarily in Algeria(13) and the USA (7). Panel (c) shows *B. anthracis* isolates, which are found globally, with notable cases in the USA (431), Tanzania (219), and Italy (81).

From the provided data on the regional distribution of *Brucella, M. tuberculosis*, and *B. anthracis*, we can draw some interesting conclusions. Firstly, the distribution of *Brucella* is relatively widespread, involving multiple countries and regions, with Italy, the USA, and Egypt reporting higher numbers of cases (Fig. 3a). This broad geographical distribution may reflect the various transmission routes and host adaptability of *Brucella*, allowing them to survive and spread in different environments and climatic conditions.

In contrast, the regional distribution of *M. tuberculosis* appears to be relatively limited, mainly concentrated in Algeria and the USA (Fig. 3b). This may be related to the transmission characteristics, host range, and differences in prevention and control measures among regions for *M. tuberculosis*. The regional distribution of *B. anthracis* also exhibits certain characteristics, with higher numbers of cases reported in the USA and Tanzania (Fig. 3c). Additionally, *B. anthracis* is present in multiple countries and regions, albeit in varying quantities. This distribution pattern may be associated with the natural environmental adaptability of *B. anthracis*, human activities, and the distribution of animal hosts.

The research significance behind these conclusions lies in the valuable information they provide about the species, host, and regional distributions of these bacterial pathogens. By delving into these characteristics, we can provide a scientific basis for formulating more effective prevention and control measures, thereby protecting the health of humans and animals. At the same time, these studies also contribute to promoting global health cooperation and jointly addressing transboundary infectious disease threats.

### Evolution of zoonosis

Phylogenetic trees serve as a cornerstone analytical tool in molecular epidemiology, enabling precise delineation of spatiotemporal dynamics in pathogen transmission chains and cross-host adaptive evolutionary trajectories through the integration of spatiotemporal evolutionary characteristics in pathogen genomes and host adaptation-related genetic variations [20]. By constructing cross-species phylogenetic frameworks for bacterial strains, this approach allows accurate reconstruction of the geographical origins of zoonotic outbreaks and directional patterns of inter-host transmission. In Evolution, we provide phylogenetic trees for *Brucella, M. tuberculosis, and B. anthracis*, along with detailed methodologies for their construction. In our implementation, we selected high-quality whole-genome sequenced strains from Zoonosis, removed redundant isolates, and reconstructed optimized phylogenetic trees for these pathogens using ITOL (https://itol.embl.de/) for visual refinement. Detailed methodological workflows have been systematically documented in the Evolution platform for user reference.

Beyond their role as topological representations of species relationships, phylogenetic trees function as critical bridges connecting genomic variations with phenotypic evolution. Their applications in infectious disease control have expanded from fundamental research to precision public health interventions. Through computational biological approaches integrating Bayesian Evolutionary Analysis Sampling Trees and maximum likelihood methods, these tools enable quantitative modelling of pathogen transmission dynamics, thereby providing evolutionary biological evidence for targeted containment strategies and emerging pathogen surveillance. This methodological framework establishes a reproducible technical paradigm for evolutionary studies of zoonotic pathogens.

### User case

To demonstrate the utility of Zoonosis, we analysed 23 sequencing datasets (sequencing datasets from NCBI), using the complete assembled genome of *B. melitensis* bv. 1 str. 16M (assembly accession: CNA0142387) as the reference genome. A systematic bioinformatics workflow was employed to elucidate genetic evolutionary relationships among the strains. The process began with single-nucleotide polymorphism (SNP) detection and core genome SNP (SNP core) extraction using Snippy v4.6.0 (https://github.com/tseemann/snippy). Whole-genome alignment generated a multiple sequence alignment (MSA) file containing genetic variation data across all samples. During this step, sequences were mapped to reference genome coordinates, and low-quality sites and recombination regions were filtered out. High-confidence SNP loci were retained to construct a genetic divergence matrix.

**Figure 4.**
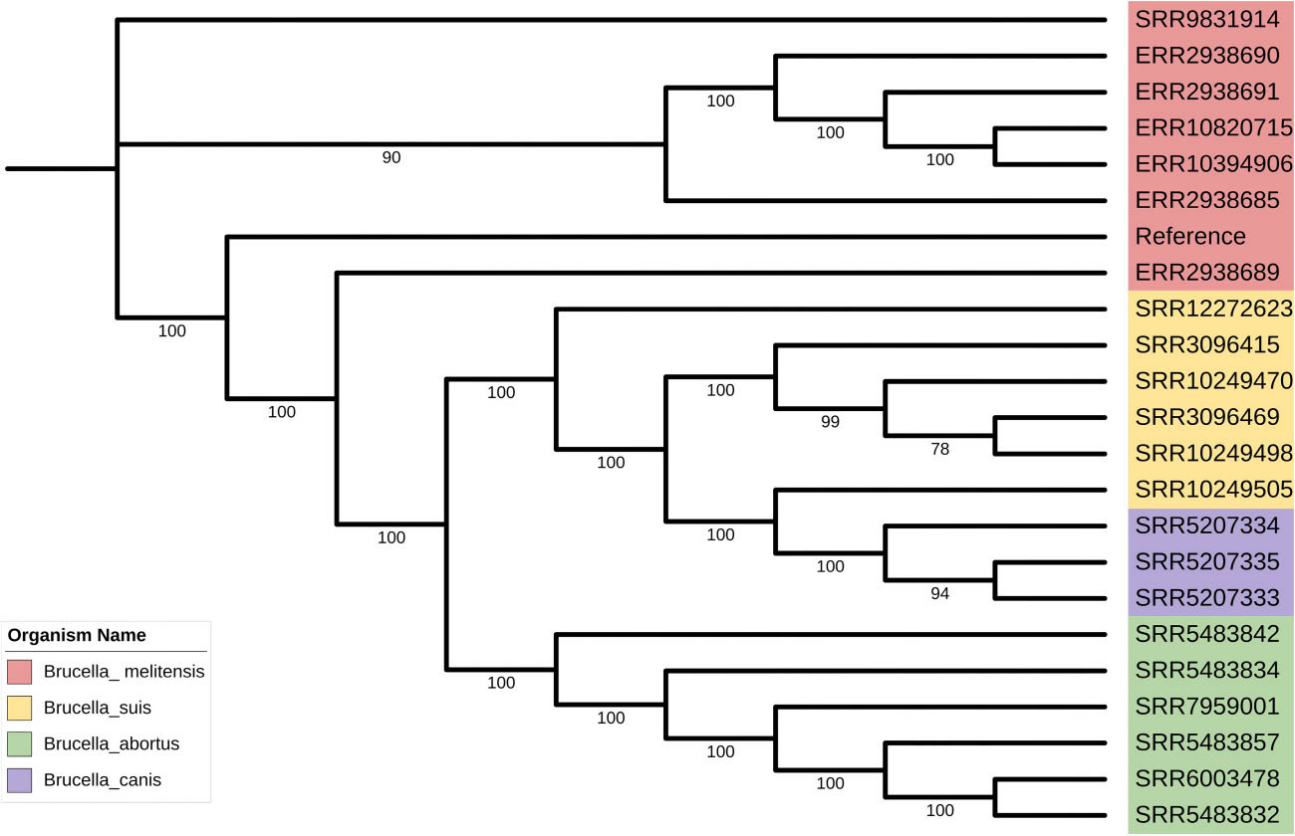
The phylogenetic relationships between various species of *Brucella*.

To visualize evolutionary relationships, FastTree [21] was used to infer a phylogenetic tree from the MSA results, which was refined and visualized using ITOL (https://itol.embl.de/) (Fig. 4). The resulting tree delineates the evolutionary relationships among *Brucella* species, with each species forming distinct monophyletic clades. These clades reflect high host-specific adaptation and reveal potential host–pathogen interactions and co-evolution. The clustering also suggests divergence in genomic, metabolic, and virulence traits among species.

Further analysis showed relatively short branch lengths within each *Brucella* species, indicating highly conserved genetic backgrounds among intraspecific strains. This conservation likely results from the genomic stability of *Brucella* and the predominance of vertical transmission, which maintains the stable inheritance of core genes. These findings provide valuable insights into the evolutionary mechanisms driving host specialization and genomic conservation in *Brucella*, offering a deeper understanding of pathogen emergence and adaptation in zoonotic systems.

## Discussion

With the acceleration of globalization and the continuous changes in the ecological environment, the threat of zoonotic diseases to human health has become increasingly significant. The development of Zoonosis (http://zoonosis.cn/zoonosis/) will focus on enhancing its capabilities to address the ever-evolving landscape of emerging infectious diseases, particularly those originating from zoonotic sources. This will involve expanding the database’s scope to incorporate data on a broader range of pathogens, including newly identified zoonotic viruses and their variants. Additionally, efforts will be made to integrate advanced analytics and machine learning algorithms, enabling more sophisticated and real-time monitoring of viral diversity, transmission patterns, and ecological dynamics. By staying at the forefront of technological advancements, Zoonosis aims to provide experts in virology, zoology, and epidemiology with cutting-edge tools for proactive surveillance and response to potential zoonotic outbreaks, thereby strengthening global public health preparedness.

Although Zoonosis provides a comprehensive platform for the integration, analysis, and visualization of zoonotic pathogen data, several limitations remain. First, the current database mainly relies on publicly available datasets, which may result in incomplete coverage for certain regions, hosts, or newly emerging pathogens. Second, the quality and consistency of metadata from different sources can vary, potentially affecting downstream analyses. Third, some zoonotic pathogens, especially those from wildlife or less-studied hosts, are underrepresented due to limited sequencing data. In addition, the integration of multi-omics data and real-time epidemiological information is still in progress. In the future, we plan to continuously expand data sources, improve metadata curation, and incorporate more advanced analytical tools to address these limitations and enhance the utility of Zoonosis for the research community.

To further solidify its position as a comprehensive resource for zoonoses and vector-borne viruses, Zoonosis will undergo strategic planning to ensure its sustainability and scalability. This includes optimizing the database architecture for efficient data storage, retrieval, and analysis, as well as implementing robust data security measures to protect sensitive information. Plans also involve expanding the user-friendly online visualization and analysis tools, making them even more intuitive and accessible to a wider audience, including policymakers and public health practitioners. Furthermore, collaborative partnerships will be fostered with international research institutions and public health organizations to facilitate data sharing and knowledge exchange, ensuring that Zoonosis remains a vital and up-to-date resource for the global community in the fight against zoonotic diseases.

## Data Availability

All the data used in this manuscript are taken from the publicly available ‘National Center for Biotechnology Information’ (NCBI) database, and all the data can be found in our database.
